# Superior osmotic stress tolerance in oilseed rape transformed with wild-type *Rhizobium rhizogenes*

**DOI:** 10.1007/s00299-024-03306-8

**Published:** 2024-08-28

**Authors:** Xuefei Chen, Henrik Lütken, Kehao Liang, Fulai Liu, Bruno Trevenzoli Favero

**Affiliations:** Section for Crop Sciences, Department of Plant and Environmental Sciences, Faculty of Science, University of Copenhagen, Taastrup, Denmark

**Keywords:** *Agrobacterium rhizogenes*, Abscisic acid, Antioxidant capacity, Osmotic potential, Polyethylene glycol, *Rhizobium rhizogenes*

## Abstract

**Key message:**

Natural transformation with *R. rhizogenes* enhances osmotic stress tolerance in oilseed rape through increasing osmoregulation capacity, enhancing maintenance of hydraulic integrity and total antioxidant capacity.

**Abstract:**

Transformation of plants using wild strains of agrobacteria is termed natural transformation and is not covered by GMO legislation in, e.g., European Union and Japan. In this study, offspring lines of *Rhizobium rhizogenes* naturally transformed oilseed rape (*Brassica napus*), i.e., A11 and B3 (termed root-inducing (Ri) lines), were investigated for osmotic stress resilience. Under polyethylene glycol 6000 (PEG) 10% (w/v)-induced osmotic stress, the Ri lines, particularly A11, had less severe leaf wilting, higher stomatal conductance (8.2 times more than WT), and a stable leaf transpiration rate (about 2.9 mmol m^−2^ s^−1^). Although the leaf relative water content and leaf water potential responded similarly to PEG treatment between the Ri lines and WT, a significant reduction of the turgid weight to dry weight ratio in A11 and B3 indicated a greater capacity of osmoregulation in the Ri lines. Moreover, the upregulation of plasma membrane intrinsic proteins genes (*PIPs*) in roots and downregulation of these genes in leaves of the Ri lines implied a better maintenance of hydraulic integrity in relation to the WT. Furthermore, the Ri lines had greater total antioxidant capacity (TAC) than the WT under PEG stress. Collectively, the enhanced tolerance of the Ri lines to PEG-induced osmotic stress could be attributed to the greater osmoregulation capacity, better maintenance of hydraulic integrity, and greater TAC than the WT. In addition, *Ri*-genes (particularly *rolA* and *rolD*) play roles in response to osmotic stress in Ri oilseed rape. This study reveals the potential of *R. rhizogenes* transformation for application in plant drought resilience.

**Supplementary Information:**

The online version contains supplementary material available at 10.1007/s00299-024-03306-8.

## Introduction

*Rhizobium rhizogenes* (formerly *Agrobacterium rhizogenes*), a gram-negative pathogenic soil bacterium, has been widely studied with great interest for its role in inducing the ‘hairy root’ syndrome (Young et al. 2001). When *R. rhizogenes* infects plants, it inserts a part, the Transfer DNA (T-DNA), of the root-inducing (Ri) plasmid into the genome of the host plant cells. This results in an increased root proliferation, termed hairy roots, at the site of infection (Chilton et al. 1982). The Ri-plasmid of agropine strains, which harbors *Ri*-genes, is divided into two regions designated as the left and right T-DNA (T_L_ and T_R_, respectively). On the T_L_, the *root oncogenic locus* (*rol*) genes are located together with other less characterized open reading frames (ORFs), while on the T_R_, the auxin homeostasis genes *aux1* and *aux2* are found among other genes (White et al. 1985).

Due to the bacterium’s capability of DNA transfer between kingdoms (Intrieri et al. 2001, Matveeva et al. 2012; Kyndt et al. 2015) (horizontal gene transfer), *R. rhizogenes*-mediated transformation (i.e., transferring *Ri*-genes into plant’s genome) has been widely used in research on breeding compact ornamental plants, particularly *Kalanchoë* as alternative to potentially hazardous chemical growth retardants (Lütken et al. 2012a, b; Hegelund et al. 2017), as well as adding a new gene pool to traditional breeding (Kuligowska et al. 2015; Cui et al. 2019; Kahraman et al. 2022). This is due to the ability of *Ri*-genes to confer compact plant growth after regeneration of entire plants from hairy roots in tissue culture (Christensen et al. 2008; Lütken et al. 2012a, b; Hegelund et al. 2018). In addition, it is also applicable in plant metabolic engineering as *Ri*-genes have shown to be potent inducers of bioactive molecules, e.g., secondary metabolites (Bulgakov 2008; Bulgakov et al. 2013; Barba-Espin et al. 2020; Martínez et al. 2020). However, increased attention has been recently centered on stress resistance in plants. For instance, enhanced oxidative defense and salinity resilience were reported in cell cultures transformed with *Ri*-genes, e.g., *rolB* and *rolC* (Kiselev et al. 2010, Shkryl et al. 2010, 2022; Bulgakov et al. 2012). These pioneering tissue culture-based studies have fueled further research aimed at exploring the association of *R. rhizogenes* with plant biotic stress resistance. In this respect, *Arabidopsis thaliana* and tomato plants overexpressing *Ri*-genes such as *rolB* exhibited enhanced fitness to drought stress (Arshad et al. 2014; Bulgakov et al. 2018; Bettini et al. 2020; Veremeichik et al. 2022). Although transgenic plants have helped to understand the function of individual *Ri*-genes, they cannot be fully compared to plants naturally transformed with *R. rhizogenes* (Ri lines), where the situation is more complex because of the synergistic activities among individual *Ri*-genes within the T-DNA (Spena et al. 1987; Shkryl et al. 2008). In this context, studies on Ri lines, based on abiotic stress resistance, are particularly important but sadly scarce at the moment, the reason primarily being the cumbersome process of regenerating plants following transformation (Hegelund et al. 2017). Root system architecture is one of the crucial factors in assessing the capacities in which plants adapt to drought (Rogers et al. 2015). Given the enhanced root system and rooting ability in Ri lines, e.g., *Kalanchoë blossfeldiana* and *Catharanthus roseus* (Suginuma et al. 1994, Lütken et al. 2012a, b; Favero et al. 2022) and even in *Ri-*genes (e.g., *rolC*) overexpressed plants such as fruit trees (Kaneyoshi et al. 1999, Koshita et al. 2002) and carnations (Zuker et al. 2001), it is expected that Ri lines would possess enhanced resistance to abiotic stress, such as drought and/or osmotic stress.

Plants have evolved sophisticated mechanisms to control water homeostasis. Under drought stress, closing of stomata controls excessive water loss from plants, and is regulated by both the rhizospheric and phyllospheric environments (Yari Kamrani et al. 2022). Abscisic acid (ABA) biosynthesis is often enhanced in plants suffering from water deficit (Song et al. 2023), acting as an important signaling modulator of the stomatal response to water availability. In addition, ABA is reported to participate in osmotic regulation by mediating the accumulation of osmolytes, thereby contributing to sustaining plant water homeostasis. On the other hand, the hydraulic signal, triggered primarily by changes in plant water status, e.g., leaf water potential (Ψ_leaf_) and leaf relative water content (RWC), is another key mechanism by which plants regulate stomatal behavior in response to water stress (Christmann et al. 2013; Tombesi et al. 2015). Moreover, root water uptake and water transport in plants are controlled by ‘water channels’ across the plasma membrane, named aquaporins (Kaldenhoff et al. 2008; Almeida-Rodriguez et al. 2011). Accumulated evidence indicates that expression of genes encoding aquaporins of the plasma membrane intrinsic proteins (*PIPs*) has a high impact on plant hydraulic conductivity, which is beneficial to water homeostasis in plants (Christmann et al. 2013; Zargar et al. 2017; Li et al. 2021). Collectively, ABA and hydraulic regulations play key roles in the maintenance of hydraulic integrity of plants under water stress.

It has been reported that the insertion of *Ri*-genes (T-DNA insertion) affects the drought response of Ri plants by altering the ABA levels (Julliard et al. 1993). In addition, numerous studies have reported that transformation with individual *Ri*-genes enhances antioxidant defense in plant tissues (Bulgakov et al. 2008, 2012; Shkryl et al. 2010). Oxidative stress is the main cause of plant damage due to drought (Ahmad et al. 2014; Ye et al. 2016), and enhanced antioxidant capacity, in turn, is considered a long-term strategy for plants to cope with drought stress (Arve et al. 2011). Based on the above, it is of interest to explore the potential of *R. rhizogenes* conferring stress resistance to plants, e.g., drought. In this study, Ri lines of oilseed rape was utilized to investigate their resistance to osmotic stress. It is worth noting that the reductions in yield in S1 Ri oilseed rape (Hegelund et al. 2018) could potentially limit the significance of studying abiotic stress resistance in Ri lines. However, the concept is that we provide pre-breeding lines to enter agricultural breeding in which some of the adverse effects (e.g., lower yield) can be diluted eventually by backcrossing. In this case, new varieties with improved stress resistance will gradually be obtained without significant loss in yield.

High-molecular-weight polyethylene glycol (PEG) treatment has been widely used to mimic plant water deficit via its osmotic effects (Verslues et al. 2006), PEG exposure can trigger rapid plant responses, including rapidly decreasing water potential and closing stomata as well as pronounced antioxidative alteration. Therefore, it has been widely applied in studies exploring effects of osmotic stress on plants, and even used for screening of plant cultivars with improved drought tolerance, e.g., tomato (Zgallai et al. 2005, Aydin et al. 2014). In the current study, the response of Ri lines of hydroponically grown oilseed rape to short-term osmotic stress was investigated by applying a PEG solution (10% (w/v)). We hypothesized that *Ri-*genes exert a positive role in mediating plant response to PEG-induced osmotic stress through effective stomatal control of water relations and enhanced antioxidant capacity, thus facilitating stress resistance in Ri lines.

## Materials and methods

### Plant materials and growth conditions

Two S1 lines (A11 and B3) of Ri oilseed rape (*Brassica napus*) winter cultivar ‘Elan’ were randomly selected as experimental materials (referred to as Ri lines in this study). Wild-type (WT) ‘Elan’ was used as the control. The primary transformant (P1) obtained from the regeneration of hairy roots into shoots later acclimatized into pots according to Hegelund et al. (2018). The plants of S1 generation were obtained by self-pollination of the P1. The *R. rhizogenes* strain used for oilseed rape transformation was A4 (ATCC43057), containing plasmid pRiA4 (Slightom et al. 1985; Jouanin et al. 1987).

Oilseed rape plants were cultivated in trays with peat (Pindstrup Substrate no. 1, Pindstrup Mosebrug A/S, Denmark) until the four-leaf stage. After washing the roots carefully, each seedling was transplanted into a 1-L container filled with Hoagland solution with a 2.0 dS m^−1^ electrical conductivity (EC) (Li et al. 2022). The growth conditions were set as 20 °C day/15 °C night and a 16 h photoperiod of natural light supplemented with artificial light with an intensity of 190–220 μmol m^−2^ s^−1^ in a greenhouse (55° 39′ 6.23″ N, 12° 17′ 31.78″ E). For all plant materials, the nutrient solution was changed every 5 days and was continuously aerated using an air compressor. The pH and EC of the nutrition solution were monitored daily: the pH was adjusted to 6.0 with 0.1 M HCl or 0.1 M NaOH; the EC was maintained at ~ 2.0 dS m^−1^.

### PEG 6000 treatments

Oilseed rape plants were grown in a hydroponic system filled with nutrient solution (Hoagland, Sigma). After 5 weeks of seed germination, each plant was transplanted to a 1-L container filled with nutrient solution containing PEG 6000 at 10% (w/v) and monitored, respectively, at 0, 2, 4, 6, and 24 h (expressed as P0, P2, P4, P6, and P24) to induce short-term osmotic stress. Stressed plants were then transferred back to PEG-free nutrient solution and monitored, respectively, at 2, 4, 6, and 24 h (expressed as R2, R4, R6, and R24) as short-term recovery. The osmotic stress level in the hydroponic solution was − 0.16 MPa for 10% PEG solution (Michel et al. 1973). Four plants were sampled at each time point, and the experiment was repeated two times displaced in time.

### Total antioxidant capacity measurement

Total antioxidant capacity (TAC) (nmol μL^−1^ FW) was assessed by the Total Antioxidant Capacity Assay kit MAK187 (Sigma) with minor modifications according to Fraser et al. (2017). The concentration of antioxidants in the sample was calculated as Trolox Equivalents. TAC was measured at P0, P24, and R24.

### Stomatal conductance measurements

At each sampling time point (P0, 2, 4, 6, 24; R2, 4, 6, 24), stomatal conductance (*g*_s_, mol m^−2^ s^−1^) was measured on the fully expanded upper leaves with a portable photosynthetic system LI-COR LI-600 (LI-Cor, NE, USA). Four plants were measured at each time point.

### Determination of plant water relations and biometric traits

**Fig. 1:**
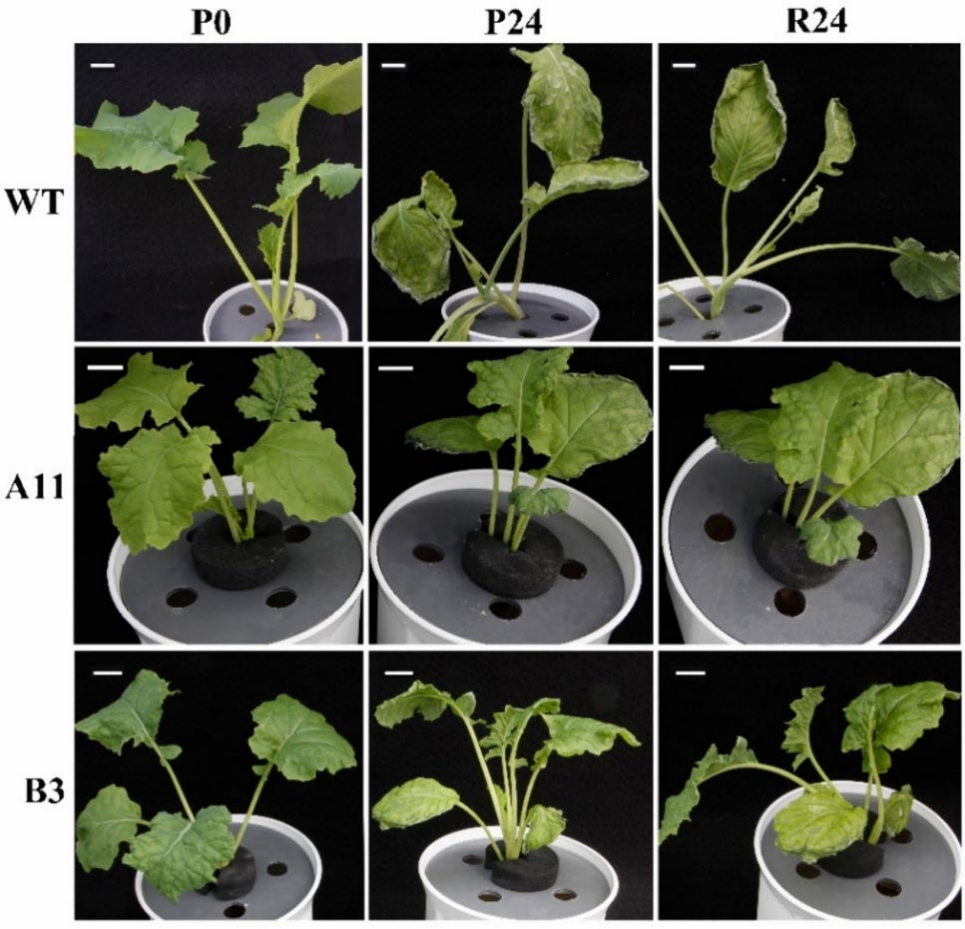
Phenotypes of A11, B3, and WT oilseed rape plants under 10% PEG (6000) treatment. Five-week-old plants were used for treatments. P0, before PEG treatmen; P24, upon 24 h of PEG stress; R24, after 24 h of stress recovery. Bar = 2 cm

Leaf area (LA, cm^2^ plant^−1^) was measured on 24 h of PEG-treated and PEG-free plants (controls) among all lines of oilseed rape by a leaf area meter (LICOR 3100). Furthermore, the water consumption per plant (WC, mL plant^−1^) within 24 h of PEG stress was determined by weighing the 1 L-containers with nutrient solutions before (W_P0_, g) and after 24 h of PEG stress (W_P24_, g). Meanwhile, oilseed rape plants growing for 24 h in the 1 L-containers without PEG were used as controls. The water density is 1 g mL^−1^ and the plant water consumption was calculated as: WC (mL) = (W_P0_–W_P24_)/1. Since the plants were grown in hydroponic containers sealed by lids (Fig. 1), the plant water consumption was assumed to be derived solely through transpiration from the leaves. Hence, the transpiration rate (*T*_r_) within 24 h of PEG stress in all lines was calculated based on LA (cm^2^ plant^−1^): *T*_r_ (mmol m^−2^ s^−1^) = (WC × 10^7^)/ (LA × 18 × 24 × 3600). Four replicates were sampled for each parameter.

At each sampling time point, a leaf was excised for the determination of midday leaf water potential (Ψ_leaf_, MPa) with a Scholander-type pressure chamber (Soil Moisture Equipment Corp., Santa Barbara, CA, USA) following the method described by Liu et al. (2006). After that, fresh weight (FW) of the excised leaves was recorded and turgid weight (TW) was obtained following a re-hydration period in distilled water for 2 h; then dry weight (DW) was measured after oven-drying at 70 ℃ for 48 h. Relative water content (RWC) was calculated as (FW – DW)/(TW – DW). Ψ_leaf_ and RWC were measured at P0, P2, P4, P6, P24, R2, R4, R6, and R24. Four replicates were sampled for each time point of each parameter.

### RNA extraction, cDNA synthesis, and quantitative real-time PCR reactions

Snap-frozen leaf and root tissues (five-week-old) were sampled at P0, P24, and R24 for RNA extraction using the RNeasy Plant Mini Kit (Qiagen, Germany). Total RNA (500 ng) was treated with DNase I Amplification Grade (Sigma Aldrich, USA), and cDNA was synthesized using the iScript cDNA Synthesis Kit (Bio-Rad, USA). The quantitative real-time (qRT)-PCR was performed using a SYBR FAST qPCR Kit (Bio-Rad) on a CFX Connect Real-Time System (Bio-Rad). The *Brassica napus Actin7* (*BnActin7*) (Hegelund et al. 2018) was selected as a reference gene for oilseed rape lines to normalize the transcript level of each investigated gene. The comparative CT (2^−ΔΔCT^) method (Livak et al. 2001) was used to calculate relative gene expression levels. Each sampling point was performed on two technical replicates and four biological replicates. Specific primers used for qRT-PCR are listed in Table S1.

### Statistical analyses

Statistical analyses were carried out using Duncan test in SPSS 21. Differences between treatments were considered as significant when *p* ≤ 0.05. Heatmaps were made by Origin 2020 and presented based on qRT-PCR data computed by 2^−ΔΔCt^.

## Results

### Plant phenotypes and expression patterns of *Ri*-genes

All oilseed rape plants showed visible signs of wilting when subjected to 10% PEG treatment for 24 h (P24), but most severely for WT, in which the whole leaves curled and hanged due to dehydration at P24 and even after 24 h of recovery (R24). In contrast, oilseed rape A11 line exhibited wilting only on the leaf edges, while B3 line showed an intermediate level of wilting compared to WT (Fig. 1).

**Fig. 2:**
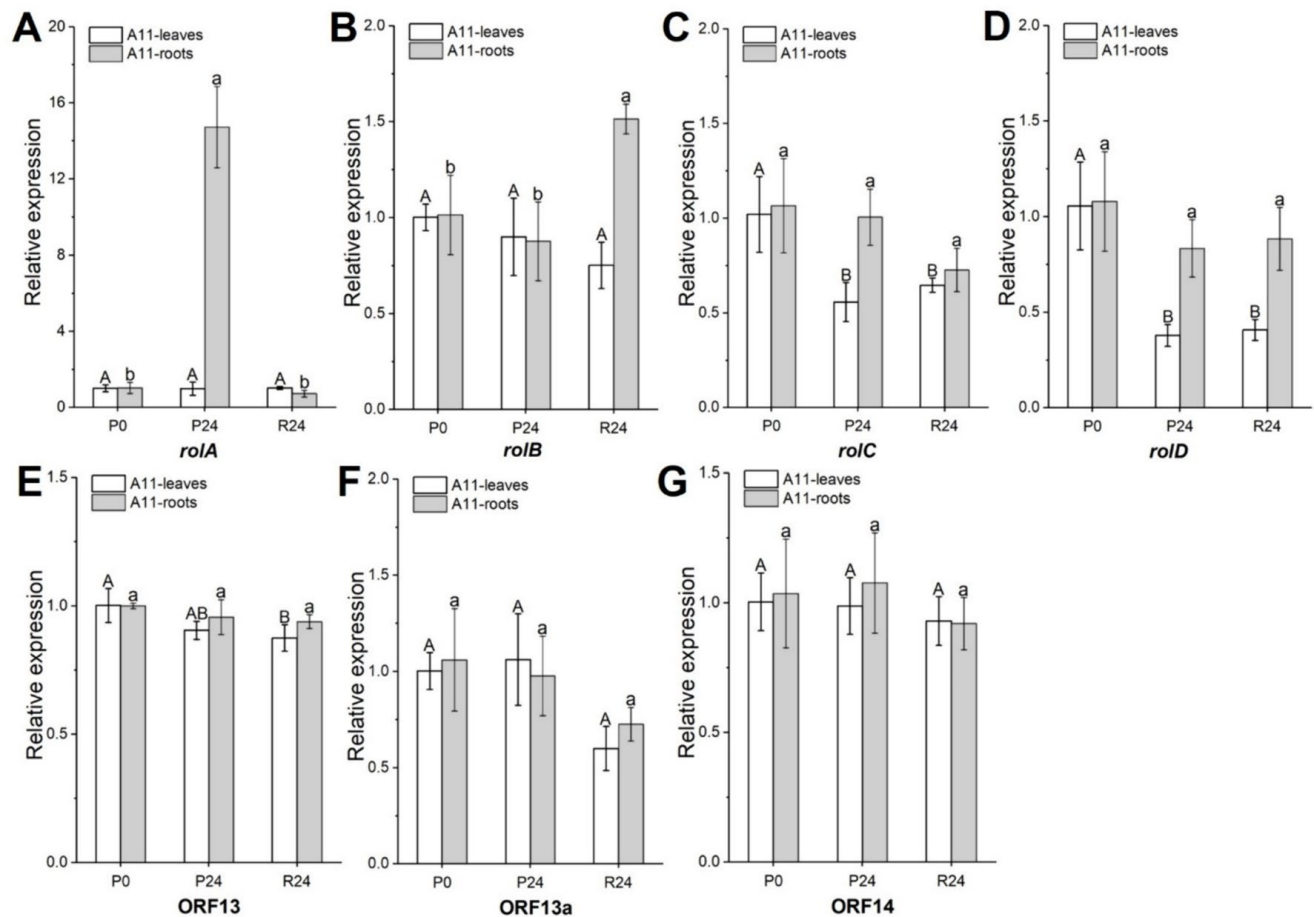
Expression patterns of *Ri*-genes in leaf and root tissues of A11 under 10% PEG (6000) treatment. P0, before PEG stress; P24, PEG stress for 24 h; R24, stress recovery for 24 h. **A** Expression patterns of *root oncogenic locus* (*rol*) *A*. **B** Expression patterns of *rolB*. **C** Expression patterns of *rolC*. **D** Expression patterns of *rolD*. **E** Expression patterns of open reading frame (ORF) 13. **F** Expression patterns of ORF13a. **G** Expression patterns of ORF14. Values are mean ± SD (*n* = 4), different letters on the top of each column indicate significance among treatments by Duncan test at *p* ≤ 0.05 level (uppercase letters indicate significance of leaves; lowercase letters indicate significance of roots)

**Fig. 3:**
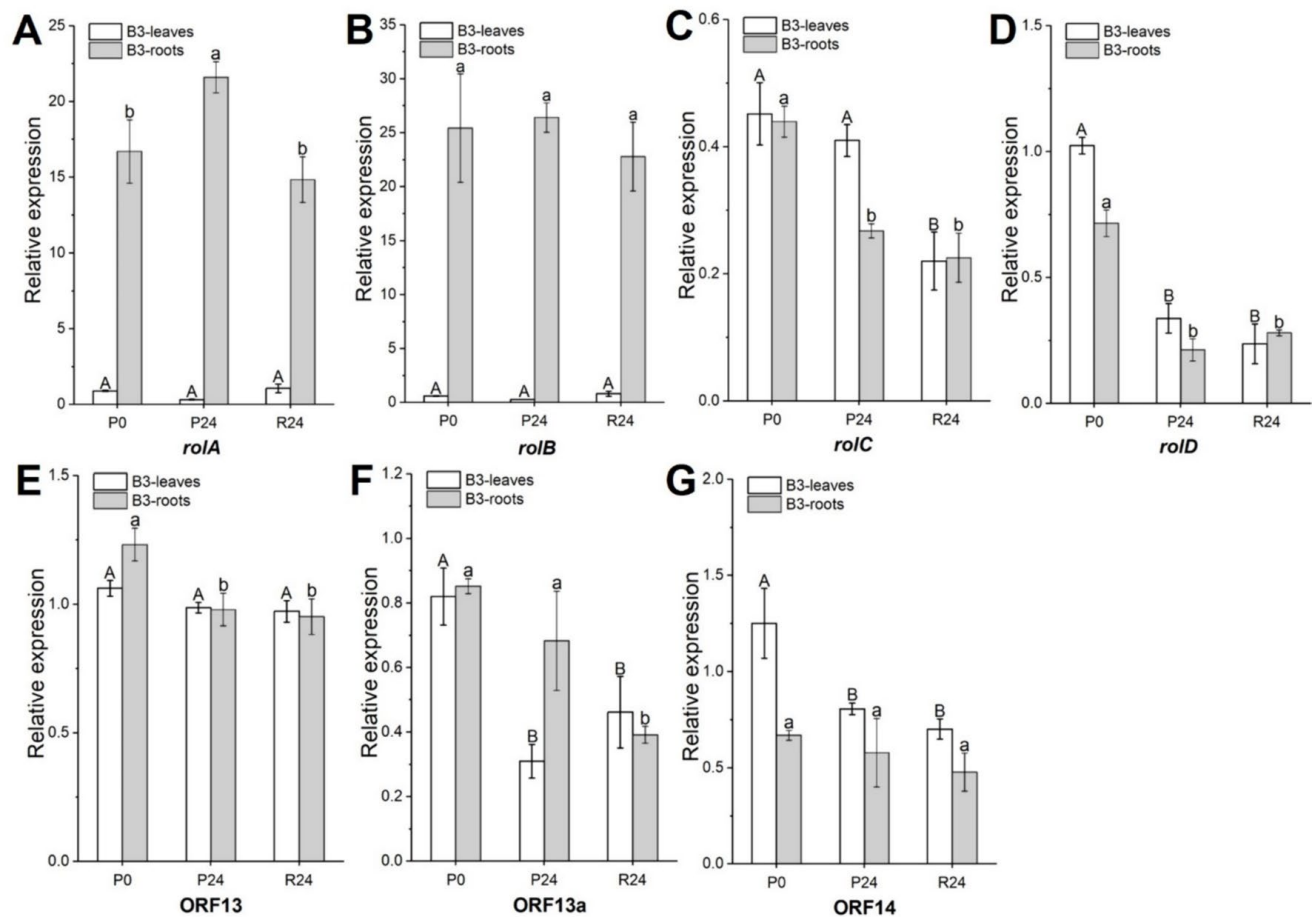
Expression patterns of *Ri*-genes in leaf and root tissues of B3 under 10% PEG (6000) treatment. P0, before PEG stress; P24, PEG stress for 24 h; R24, stress recovery for 24 h. **A** Expression patterns of *root oncogenic locus* (*rol*) A. **B** Expression patterns of *rolB*. **C** Expression patterns of *rolC*. **D** Expression patterns of *rolD*. **E** Expression patterns of open reading frame (ORF) 13. **F** Expression patterns of ORF13a. **G** Expression patterns of ORF14. Values are mean ± SD (*n* = 4), different letters on the top of each column indicate significance among treatments by Duncan test at *p* ≤ 0.05 level (uppercase letters indicate significance of leaves; lowercase letters indicate significance of roots)

The expression patterns of seven selected *Ri*-genes, which were well-characterized, were investigated in leaf and root tissues of A11 and B3 lines, respectively (Figs. 2 and 3). For the A11 line, following exposure to PEG stress and subsequent recovery, expression levels of *rolC*, *rolD*, and ORF13 were downregulated after 24 h of PEG treatment (at P24) by 2.0-, 3.3-, and 1.1-fold, respectively, in the leaves of A11 compared to those at P0; these expression levels after 24 h of recovery (R24) remained consistent with those at P24 (Fig. 2C–E). In the root tissues, only the expression levels of *rolA* were increased in A11 roots at P24, being 14.7-fold higher than that at P0 (before PEG stress), and decreased dramatically to its original level (as P0) after stress recovery (at R24) (Fig. 2A). However, the remaining *Ri*-genes were expressed relatively stable in the A11 roots during PEG stress and recovery (Fig. 2).

For the leaf tissues of B3 line, the expression levels of *rolD*, ORF13a, and ORF14 were decreased by 3.3-, 2.6-, and 1.6-fold, respectively, at P24 compared to those at P0, and maintained at the same levels as P24 thereafter (at R24) (Fig. 3D, F, G). In the roots of B3, the expression of *rolA* was 1.4-fold upregulated, whereas *rolC*, *rolD*, and ORF13 were 1.6-, 3.5- and 1.3-fold downregulated, respectively, at P24 compared to those at P0 (Fig. 3C, D, E). Overall, *rolA* and *rolB* were highly expressed in B3 roots both before and after PEG treatment compared with those in leaves (Fig. 3A, B).

### Plant water relation characteristics and turgid weight and dry weight ratio (TW/DW)

**Fig. 4:**
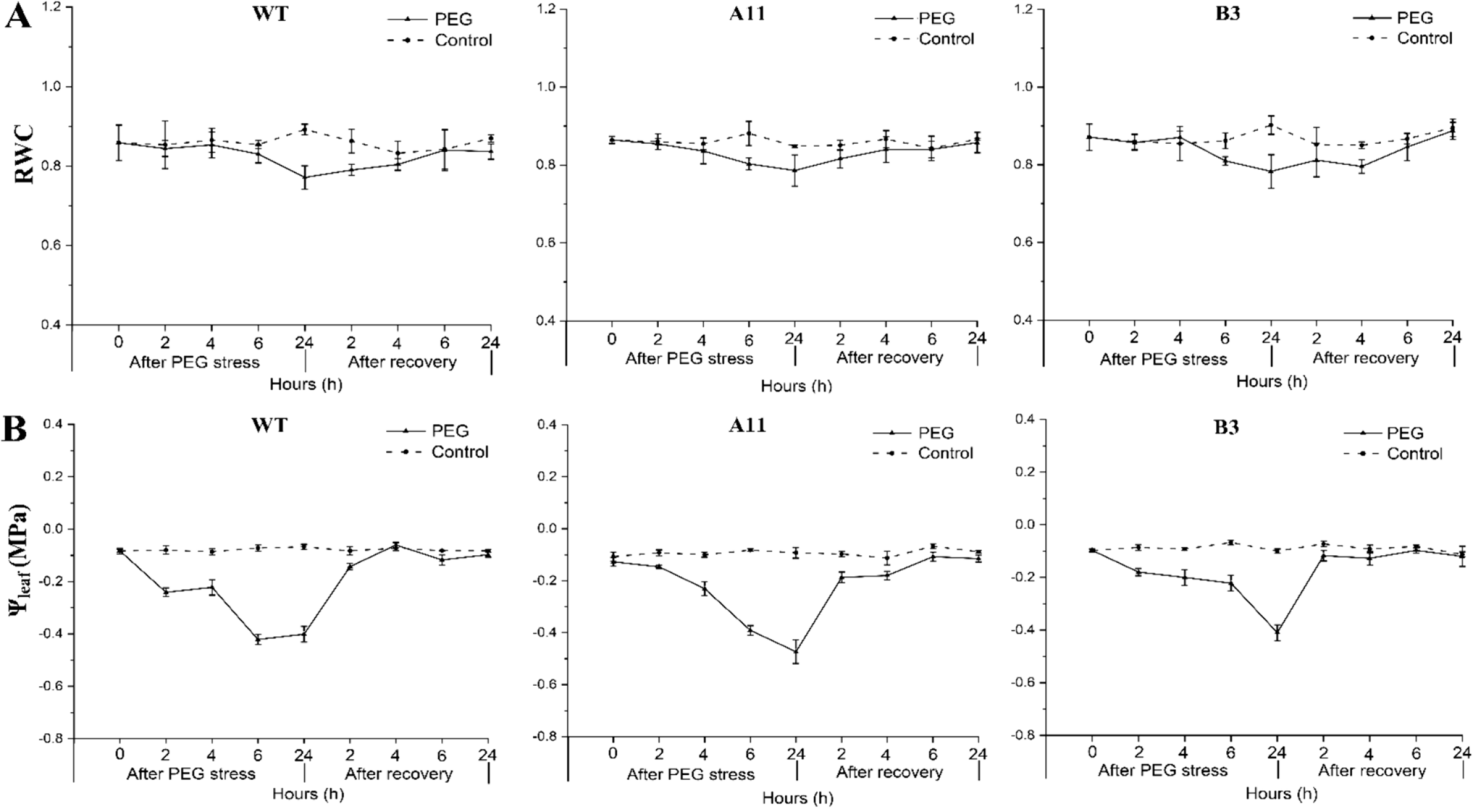
Relative water content (RWC) and midday leaf water potential (Ψ_leaf_) of oilseed rape lines during 10% PEG (6000) stress and recovery. **A** RWC of A11, B3, and WT lines. **B** Ψ_leaf_ of A11, B3, and WT lines. PEG indicates plants exposed to 10% PEG; control indicates plants grown without PEG

**Fig. 5:**
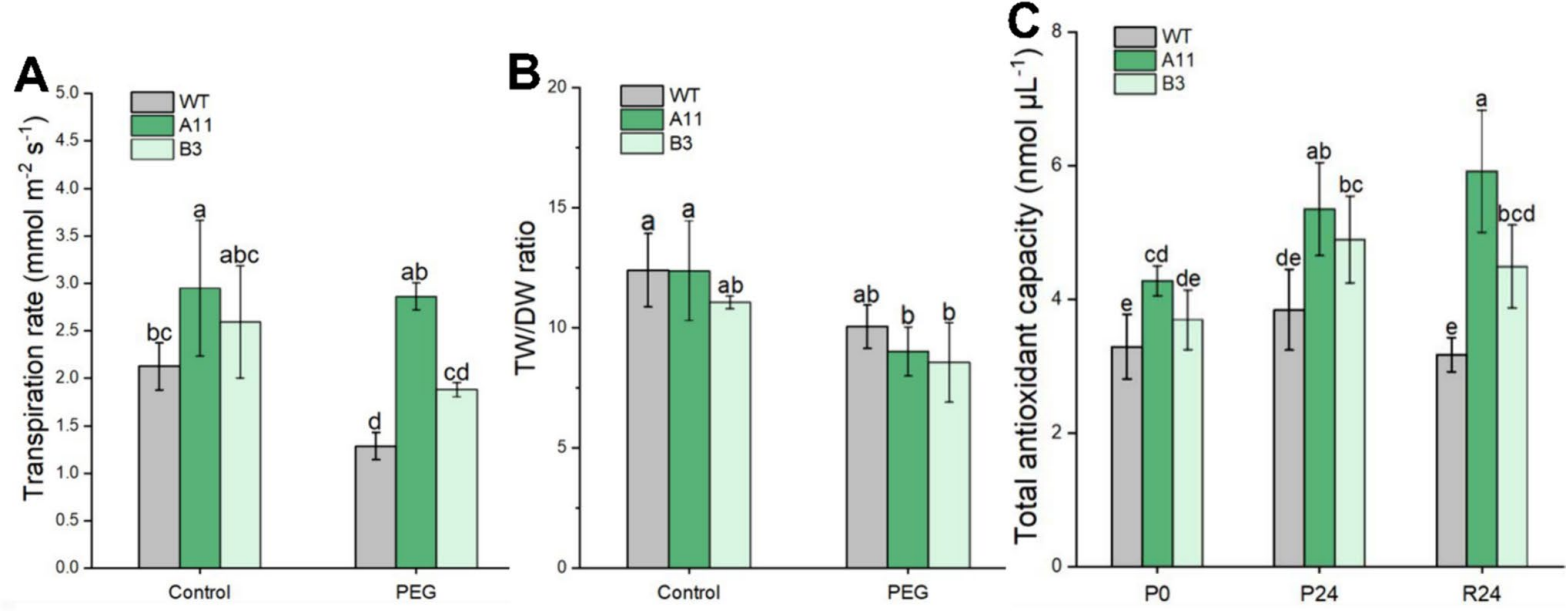
Transpiration rate (*T*_r_), turgid weight and dry weight ratio (TW/DW), and total antioxidant capacity (TAC) in leaves of oilseed rape under 10% PEG (6000) treatment. *T*_r_ (**A**) and TW/DW (**B**) within 24 h of PEG stress (PEG) and without PEG stress (control). **C** TAC; P0, before PEG treatment; P24, upon 24 h of PEG stress; R24, after 24 h of stress recovery. Values are mean ± SD (*n* = 4), different letters on the top of each column indicate significance among genotypes by Duncan test at *p* ≤ 0.05 level

Regarding the average values of RWC and Ψ_leaf_, there was no significant difference among the three genotypes before and after PEG stress (Table S2). Moreover, RWC and Ψ_leaf_ in the three genotypes showed similar development throughout the PEG treatment, i.e., a distinct decrease upon stress and recovered levels after stress recovery (Fig. 4). In addition, the transpiration rate (*T*_r_) in all lines within 24 h of PEG stress was calculated based on their leaf area and water consumption (Fig. 5A and Fig. S1). For PEG-treated plants, *T*_r_ was significantly higher in A11 (2.9 ± 0.14 mmol m^−2^ s^−1^) than in B3 (1.9 ± 0.08 mmol m^−2^ s^−1^) and WT (1.3 ± 0.14 mmol m^−2^ s^−1^). However, for PEG-free plants, no significant difference in *T*_r_ was noticed between A11 (2.9 ± 0.71 mmol m^−2^ s^−1^) and B3 (2.6 ± 0.60 mmol m^−2^ s^−1^), or between B3 and WT (2.1 ± 0.25 mmol m^−2^ s^−1^). Besides, within 24 h of PEG stress, there was no notable difference in *T*_r_ between PEG-treated and PEG-free plants of Ri lines. However, *T*_r_ was significantly decreased in PEG-treated WT plants compared to its controls (Fig. 5A).

The turgid weight and dry weight ratio (TW/DW) of leaves were measured after 24 h of PEG-treated and non-PEG conditions among all oilseed rape lines (Fig. 5B). The TW/DW of all lines of oilseed rape ranged from 11.1 to 12.4 in non-PEG conditions and decreased after 24 h of PEG stress to 8.6–10.0. However, only the reduction of TW/DW in A11 was statistically significant.

### Total antioxidant capacity (TAC) in leaf tissues

The TAC in leaves of three genotypes of oilseed rape is shown in Fig. 5C. Before PEG exposure, A11 had the highest TAC (4.3 ± 0.2 nmol μL^−1^), which was 1.3 and 1.2 times higher than in WT (3.3 ± 0.5 nmol μL^−1^) and B3 (3.7 ± 0.4 nmol μL^−1^), respectively. Upon 24 h of PEG stress, the TACs of A11 and B3 increased significantly on average by 1.3-fold compared with those at P0. However, TAC in WT was not significantly induced by 24 h of PEG stress.

### Plant stomatal conductance (*g*_s_)

**Fig. 6:**
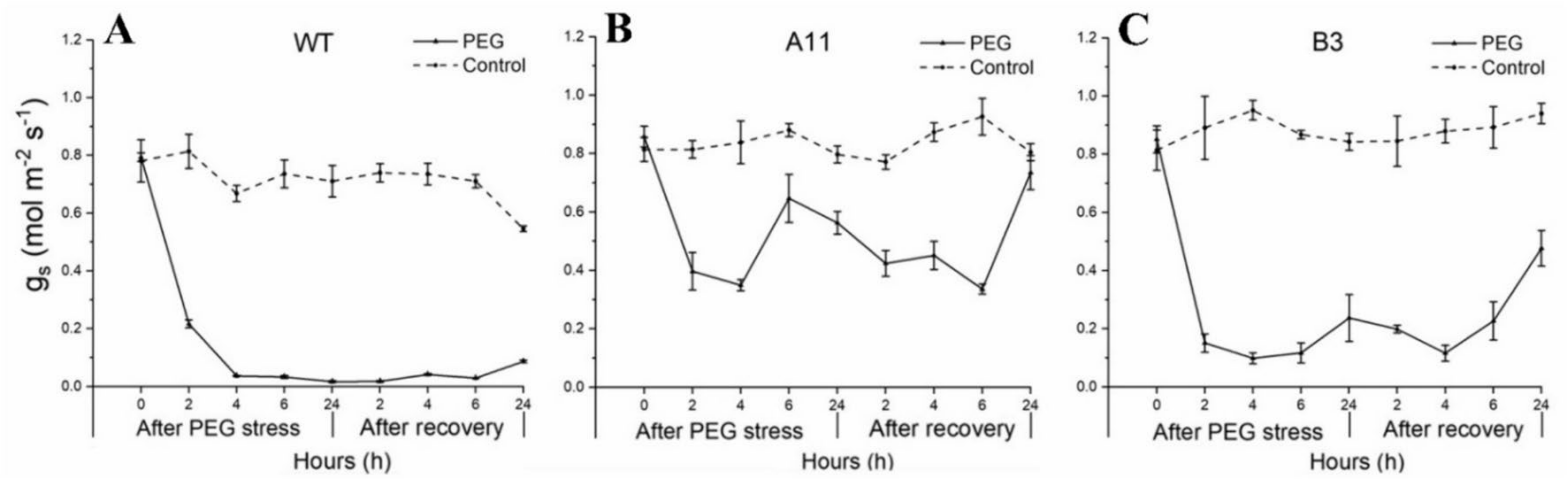
Stomatal conductance (*g*_s_) of oilseed rape lines during 10% PEG (6000) stress and recovery. **A** *g*_s_ of WT line. **B** *g*_s_ of A11 line. **C** *g*_s_ of B3 line. PEG indicates plants exposed to 10% PEG, control indicates plants grown without PEG

The average *g*_s_ of plants before and after PEG treatment is shown in Table S2. Under well-watered conditions (P0), there was no significant difference in the average *g*_s_ among the oilseed rape lines. Besides, A11 exhibited the highest average *g*_s_, being 0.49 ± 0.14 mol m^−2^ s^−1^; in contrast, WT showed the lowest average *g*_s_, being 0.06 ± 0.06 mol m^−2^ s^−1^, when exposed to PEG stress and recovery. In addition, all oilseed rape lines exhibited a sharp decrease in *g*_s_ within the first 2 h upon PEG treatment, with A11 showing the lowest reduction (0.40 ± 0.06 mol m^−2^ s^−1^) followed by WT (0.22 ± 0.01 mol m^−2^ s^−1^) and B3 (0.17 ± 0.03 mol m^−2^ s^−1^) (Fig. 6A–C). Only A11 resumed the initial *g*_s_ at the end of the recovery period (R24), while B3 reached 50% and WT 15% of its respective control plants kept in PEG-free nutrient solution.

### Expression of plasma membrane intrinsic proteins genes (*PIPs*)

**Fig. 7:**
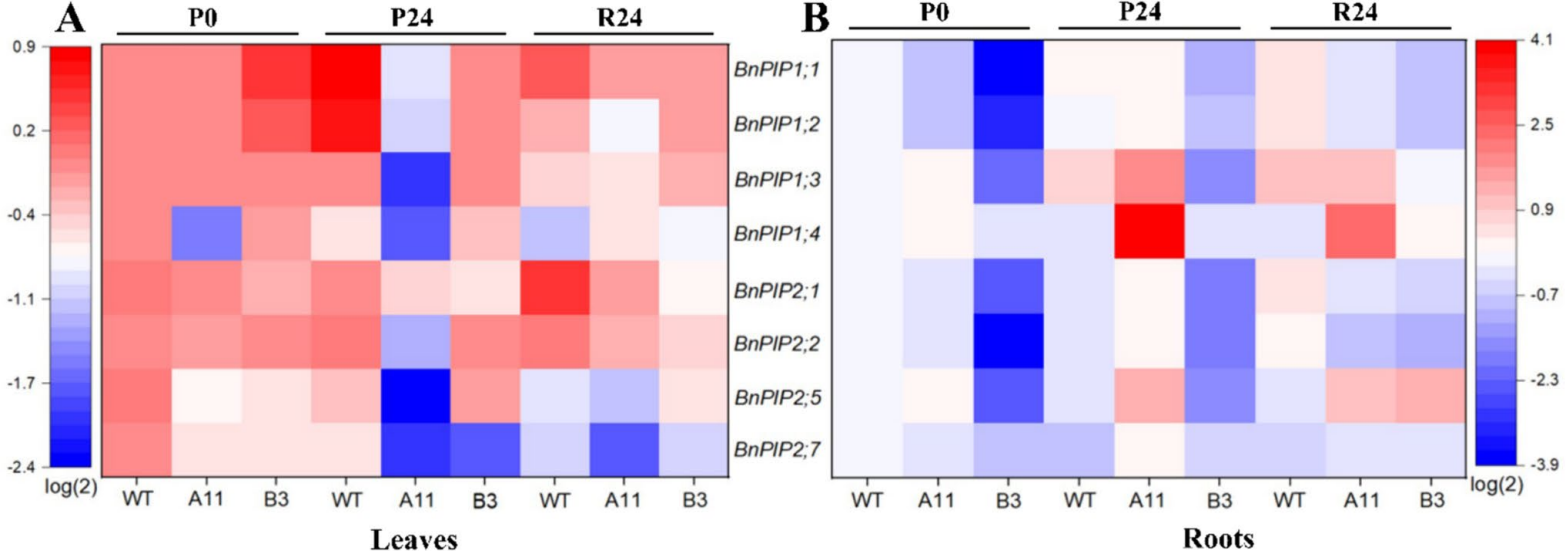
Expression patterns of plasma membrane intrinsic proteins genes (*PIPs*) in leaf and root tissues of oilseed rape lines under 10% PEG (6000) stress and recovery. *n* = 4. **A** Expression patterns of *PIPs* in leaf tissues of A11, B3, and WT. **B** Expression patterns of *PIPs* in root tissues of A11, B3, and WT. P0, before PEG treatment; P24, upon 24 h of PEG stress; R24, after 24 h of PEG recovery. *Bn* indicates *Brassica napus*. *PIP1;1* plasma membrane intrinsic protein 1;1, *PIP1;2* plasma membrane intrinsic protein 1;2, *PIP1;3* plasma membrane intrinsic protein 1;3, *PIP1;4* plasma membrane intrinsic protein 1;4, *PIP2;1* plasma membrane intrinsic protein 2;1, *PIP2;2* plasma membrane intrinsic protein 2;2, *PIP2;5* plasma membrane intrinsic protein 2;5, *PIP2;7* plasma membrane intrinsic protein 2;7

Under PEG stress and recovery, expression patterns of major genes of *PIPs*, including *BnPIP1;1*, *BnPIP1;2*, *BnPIP1;3*, *BnPIP1;4*, *BnPIP2;1*, *BnPIP2;2*, *BnPIP2;5*, and *BnPIP2;7*, were detected in leaf and root tissues of oilseed rape responding to water deficit (Fig. 7). In leaves, differences in expression profiles of *PIPs* between Ri lines and WT were detected before PEG exposure; especially for *BnPIP1;4*, *BnPIP2;5*, and *BnPIP2;7*, which were expressed significantly lower by 1.5–3-fold in A11 and B3 than in WT leaves at P0 (Fig. 7A). Upon PEG stress and recovery, the expression of *PIPs* was generally downregulated or remained at the same levels as those at P0 in A11 and B3 leaves. In comparison, the expression of *PIPs* did not regularly respond to PEG in WT leaves, with some *PIPs* being upregulated but others downregulated in expression. On the other hand, in the roots (Fig. 7B), all the *PIPs* were expressed dramatically lower in B3 than in WT before PEG exposure; especially for the *BnPIP1;1*, *BnPIP1;2*, and *BnPIP2;2*, which were expressed lower by 15-, 10-, and 13-fold, respectively, in B3 roots than that of WT at P0. However, in the A11 roots, the expression of all *PIPs* was either slightly lower or the same as that in WT roots before stress. Upon 24 h of PEG treatment, most of the *PIPs* maintained the same levels in WT roots at P0 and P24 and were significantly upregulated by about 1.5–2-fold at R24 as compared to the P24. In B3 roots, three *PIPs* were significantly induced by PEG treatment, with the expression of *BnPIP1;1* and *BnPIP1;2* significantly increased at P24 and expression of *BnPIP2;5* particularly upregulated at R24. In addition, expressions of all *PIPs* were obviously induced by PEG in A11 roots, with all *PIPs* expression increased by 1–5-fold at P24 and subsequently decreasing by about 1.5-fold at R24.

### PCA analysis of physiological parameters and expression of genes after 24 h PEG stress

**Fig. 8:**
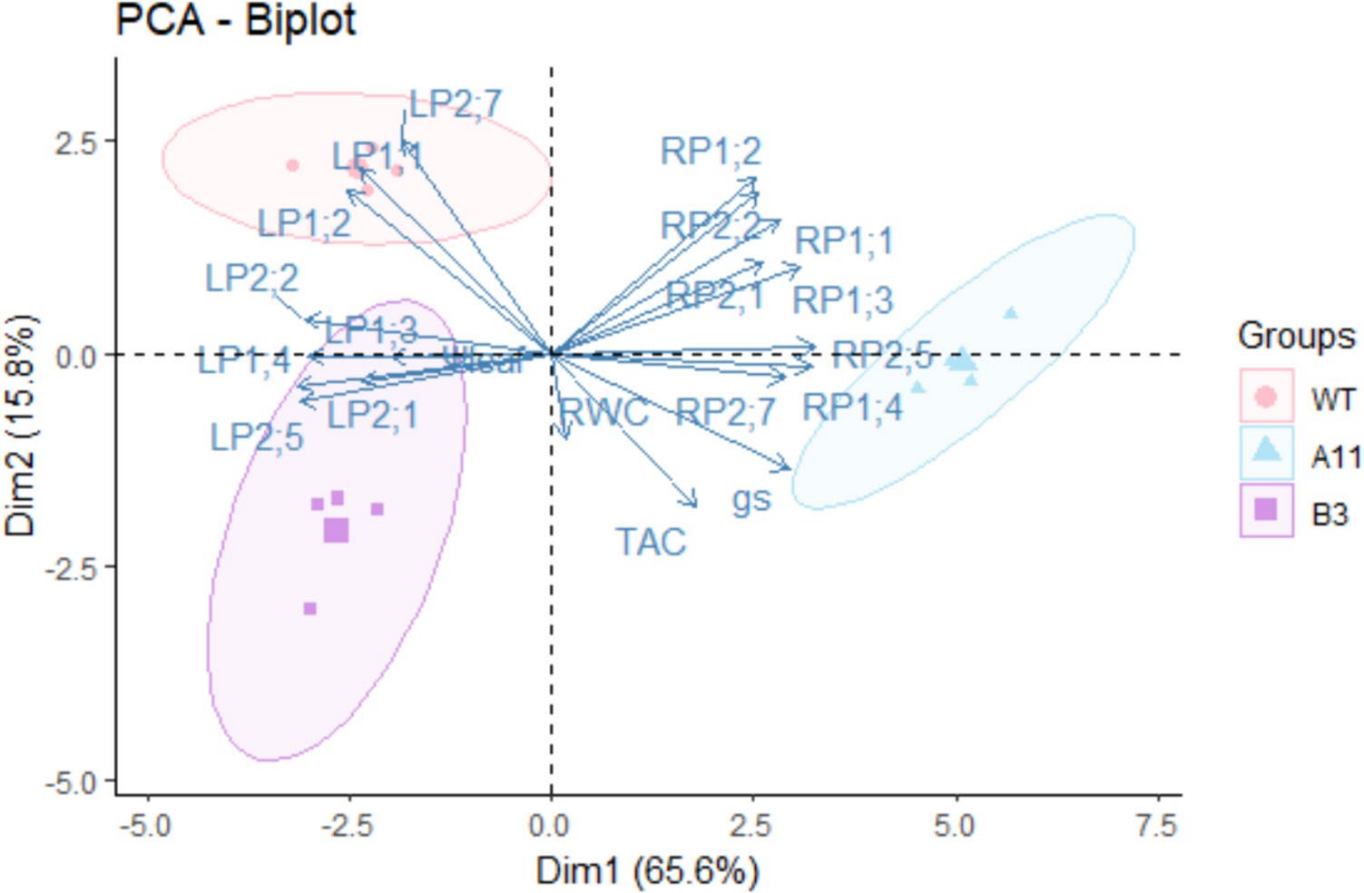
Principal component analysis of oilseed rape A11, B3, and WT lines after 24 h of 10% PEG (6000) stress. Parameters used for PCA include stomatal conductance (*g*_s_), leaf total antioxidant capacity (TAC), leaf water potential (Ψ_leaf_), leaf relative water content (RWC), and expression of plasma membrane intrinsic proteins genes (*PIPs*) including leaf *PIPs* (LP) and root *PIPs* (RP)

At P24, PCA plots of physiological parameters including *g*_s_, TAC, RWC, and Ψ_leaf_, and expression of *PIPs* (leaf/root *PIPs*) were depicted (Fig. 8). Overall, PCA showed clear group formation related to A11, B3, and WT, and explained 81.4% variation for them. The expressions of leaf *PIPs* were clustered in the left part of the PCA plot and the opposite for all root *PIPs*, clustering with A11. Moreover, WT showed a strong correlation with leaf *PIP1;1*, *PIP1;2*, and *PIP2;7*.

## Discussion

High-molecular-weight polyethylene glycol (PEG) treatment has been widely used to mimic plant water deficit via osmotic effects (Verslues et al. 2006). When plants encounter severe osmotic stress, the root water uptake is constrained, thus curtailing water supply to shoot, resulting in wilting of leaves (Verslues et al. 2006; Christmann et al. 2013). In this study, the Ri lines exhibited greater resistance to PEG-induced osmotic stress as exemplified by the less severe symptoms of leaf wilting in the A11 and B3 lines compared to the WT (Fig. 1). Besides, *T*_r_ in both Ri lines showed no significant changes in PEG-treated plants compared to PEG-free plants. However, this was not the case in WT where a notable drop in *T*_r_ was found in the PEG-treated group as compared to the PEG-free plants (Fig. 5A). These results suggest that the water uptake and transpiration rate in the A11 and B3 lines had not been severely affected by PEG stress in relation to the WT plants. Interestingly, the expression of gene encoding major PIP aquaporins (*PIPs*) in roots was also upregulated in the Ri lines, particularly A11 (Fig. 7B), which would imply enhanced root hydraulic conductance, hence facilitating water uptake (Christmann et al. 2013; Zargar et al. 2017; Li et al. 2021). This was corroborated in the PCA plots where nearly all root *PIPs* were clustered to A11 lines after PEG stress (Fig. 8). On the other hand, the expression of *PIPs* was generally downregulated in leaves of A11 and B3 under osmotic stress, suggesting a reduced leaf hydraulic conductance that would restrict leaf water loss (Johnson et al. 2014; Fang et al. 2019). Consequently, an enhanced root water uptake capacity and a restricted water loss from leaves would result in a better maintenance of hydraulic integrity, thus less severe wilting symptoms in the Ri lines under osmotic stress (Fig. 1). Nevertheless, *T*_r_ in PEG-treated A11 and B3 lines did not significantly decrease as compared to the PEG-free plants, which seems to contradict the presumed reduction in leaf water loss due to downregulated *PIPs*. This could be due to the enhanced root water uptake that sustained the transpirational water loss, which ultimately maintained the water homeostasis in the plant. However, this was not supported by the results of leaf RWC and Ψ_leaf_ (Fig. 4), where the Ri lines responded similarly to PEG as WT. Nevertheless, the greater reduction of TW/DW by PEG stress in the Ri lines as compared to the WT indicated a greater capacity of osmoregulation (Jensen et al. 1996, 2000, Liu et al. 2002), which would help to sustain the leaf turgor. Collectively, low TW/DW, hence a high capacity of osmoregulation, could sustain a potential gradient for water uptake and turgor maintenance during water stress (Yan et al. 2017). That is probably the reason that less severe wilting symptoms and higher *g*_s_ were evident in Ri lines as compared to WT (Fig. 1 and Fig. 6), even though the reduction of leaf water potential under stress was similar among all genotypes (Fig. 4 and Table S2). Therefore, it follows the enhanced resilience of the Ri lines to osmotic stress could be attributed to the better maintenance of hydraulic integrity and greater osmoregulation capacity than the WT.

Comparing to Ri lines, WT had significant lower TAC after PEG stress and recovery (Fig. 5C), which suggests a severe oxidative damage in leaves (Li et al. 2020; Asad et al. 2021), as exemplified by WT’s severely wilted leaves after osmotic stress (Fig. 1). However, the TAC was notably increased by PEG stress in Ri lines (Fig. 5C). The antioxidant capacity was reported to increase with the severity of drought in different oilseed rape cultivars (Abedi et al. 2010) as well as other species, such as *Oudeneya africana* (Talbi et al. 2020) and tomato plants (Zhou et al. 2019). Therefore, it can be suggested that enhanced protection against oxidative stress in Ri lines could be one of strategies to respond to osmotic stress.

Individual *Ri*-genes, such as *rolB* and *rolC*, have been widely reported to be involved in abiotic stress resistance (e.g., drought) in plants or plant tissues (Shkryl et al. 2010; Veremeichik et al. 2022). Here we analyzed the transcript levels of representative *Ri*-genes in Ri plants, to verify how they respond to the osmotic stress caused by PEG exposure. After PEG stress (P24) and recovery (R24), the expression levels of all tested *Ri*-genes, including *rolA*-*D*, ORF13, ORF13a, and ORF14, fluctuated to different degrees in leaves and roots of A11 and B3 lines (Figs. 2 and 3). This suggests the involvement of the *Ri*-genes in the osmotic stress response of oilseed rape. Notably, in the roots of A11 and B3 lines, expression of *rolA* was dramatically upregulated by PEG stress (Figs. 2A and 3A), which was consistent with the upregulation of *PIPs* expression in roots of Ri lines under osmotic stress. Moreover, the expression of *rolD* in leaves of A11 and B3 was significantly downregulated by PEG stress (Figs. 2D and 3D), which coincided with the downregulation of *PIPs* expression in leaves of Ri lines under osmotic stress. However, more research is merited to reveal the mechanisms behind these associations. Taken together, these results suggest that *rolA* and *rolD* genes may play roles in response to osmotic stress in Ri oilseed rape.

## Conclusion

In this study, the Ri lines of A11 and B3 possess enhanced tolerance to short-term osmotic stress compared with WT plants. The greater osmoregulation capacity, better maintenance of hydraulic integrity, and greater antioxidant capacity in Ri lines could be the strategies to better cope with osmotic stress. In addition, *rolA* and *rolD* genes may play roles in response to osmotic stress in Ri oilseed rape. This finding reveals the potential of *R. rhizogenes* transformation for application in plant drought resilience.

## Supplementary Information

Below is the link to the electronic supplementary material.

Supplementary file1 (PDF 459 KB)

Supplementary file2 (DOCX 17 KB)

Supplementary file3 (DOCX 15 KB)

## Data Availability

All data supporting the findings of this study are available within the paper and within its supplementary data published online.
