# Supplementary material for: Superior osmotic stress tolerance in oilseed rape transformed with wild-type Rhizobium rhizogenes
Source: Plant Cell Rep. 2024 Aug 28;43(9):223. doi: 10.1007/s00299-024-03306-8 (PMC11358183; doi:10.1007/s00299-024-03306-8)
Supplement: Supplementary file 1 — Supplementary file1 (PDF 459 KB) [file 299_2024_3306_MOESM1_ESM.pdf]

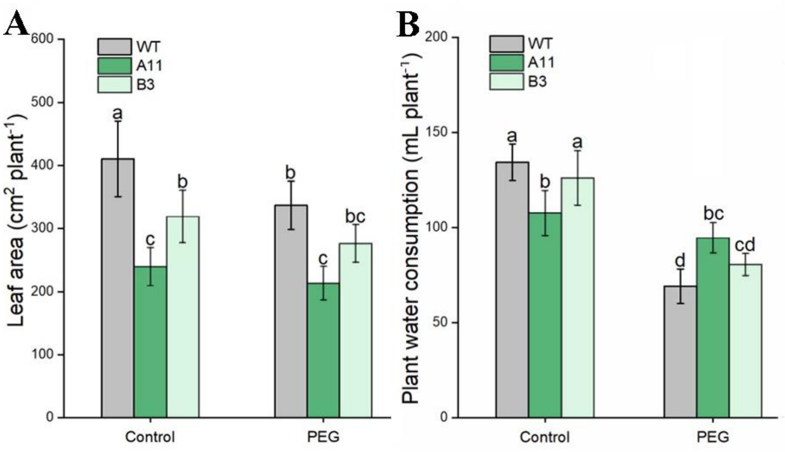

Figure S1. Plant leaf area and water consumption under 10% PEG (6000) and non-PEG conditions. Plant leaf area (A) and water consumption (B) within 24 h of PEG stress (PEG) and without PEG stress (control). Values are mean ± SD (n = 4), different letters on the top of each column indicate significance among genotypes and treatments by Duncan test at $P \leq 0.05$ level.
