# Supplementary material for: Superior osmotic stress tolerance in oilseed rape transformed with wild-type Rhizobium rhizogenes
Source: Plant Cell Rep. 2024 Aug 28;43(9):223. doi: 10.1007/s00299-024-03306-8 (PMC11358183; doi:10.1007/s00299-024-03306-8)
Supplement: Supplementary file 2 — Supplementary file2 (DOCX 17 KB) [file 299_2024_3306_MOESM2_ESM.docx]

| **Gene name** | | **Forward primer (5'-3')** | | **Reverse primer (5'-3')** |
| --- | --- | --- | --- | --- |
| *Ri*-genes | | | | |
| *rolA* | ﻿CCAATCTGAGCACCACTCCT | | ﻿AATCCCGTAGGTTTGTTTCG | |
| *rolB* | GATATCCCGAGGGCATTTTT | | GAATGCTTCATCGCCATTTT | |
| *rolC* | CAATAGAGGGCTCAGGCAAG | | CCTCACCAACTCACCAGGTT | |
| *rolD* | GCGAAGTGGATGTCTTTGG | | ﻿TTGCGAGGTACACTGGACTGA | |
| ORF13 | TGTCGATGATTTTCGTTGGA | | ATTTCCGCATTGTTGACCTC | |
| ORF13a | GGCTTGTAACGGACCTTGTG | | CGACGGGGAAATATGTTCTT | |
| ORF14 | CGCAAAAGTAACCTCGCTTC | | CACTCTGATCCTGTGGCTGA | |
| Plasma membrane intrinsic proteins genes (*PIPs*) | | | | |
| *BnPIP1;1* | | CACTGTTTTGACCGTCATGG | | TCCAAGACCACTTCCTTTGG |
| *BnPIP1;2* | | CTTGCTTCCTGGTCCTTCTG | | GGCTCCACCTCCTAGAGCTT |
| *BnPIP1;3* | | CTTTCGGTGGCATGATCTTT | | AGCGGAGAAGACGGTGTAGA |
| *BnPIP1;4* | | ACATCAGCTCAGTCCGACAA | | CCTAGCCAAGAACAGACCGA |
| *BnPIP2;1* | | CGAGTTCGTAGCCACTCTCC | | AACCGCTCTAACCAGCGATA |
| *BnPIP2;2* | | GTGACGTTCGGCTTGTTCTT | | AGTGGCCAAGTGTACCATGA |
| *BnPIP2;5* | | CCCTTTACCCTGACCAGTGT | | CGGAGAAGACGGTGTAGAC |
| *BnPIP2;7* | | ACGGCGTCGGTTTACTCG | | ACCACAAATGGCTCCAAG |
| Reference gene | | | | |
| *BnActin7* | | TGGAGAAGAACTACGAGCTAC | | TTGAACCACCACTGAGGAC |

**Table S1.** **Primer sequences for qRT-PCR analysis**
