# Supplementary material for: Superior osmotic stress tolerance in oilseed rape transformed with wild-type Rhizobium rhizogenes
Source: Plant Cell Rep. 2024 Aug 28;43(9):223. doi: 10.1007/s00299-024-03306-8 (PMC11358183; doi:10.1007/s00299-024-03306-8)
Supplement: Supplementary file 3 — Supplementary file3 (DOCX 15 KB) [file 299_2024_3306_MOESM3_ESM.docx]

**Table S2. Average values of *g*_s_, RWC, and Ψ_leaf_ of oilseed rape lines before and after 10% PEG (6000) treatment**

|  | **Well-watered condition** | | | **PEG stress and recovery** | | |
| --- | --- | --- | --- | --- | --- | --- |
|  | **WT** | **A11** | **B3** | **WT** | **A11** | **B3** |
| *g*_s_ (mol m^-2^ s^-1^) | 0.79±0.08 | 0.82±0.08 | 0.89±0.04 | 0.06±0.06c | 0.49±0.14a | 0.22±0.15b |
| RWC | 0.86±0.02 | 0.86±0.01 | 0.87±0.02 | 0.82±0.03 | 0.83±0.03 | 0.83±0.04 |
| Ψ_leaf_ (Mpa) | -0.08±0.01 | -0.09±0.01 | -0.09±0.01 | -0.21±0.14 | -0.23±0.13 | -0.18±0.10 |

Well-watered condition indicates before PEG treatment (P0); PEG stress and recovery indicates after PEG stress and recovery, i.e. P2, P4, P6, P24, R2, R4, R6 and R24. *g*_s_, stomatal conductance; RWC, relative water content; Ψ_leaf_, midday leaf water potential. Different letters indicate significance among genotypes by Duncan test at *P* ≤ 0.05 level.
